# The impact of project ECHO on physician preparedness to treat opioid use disorder: a systematic review

**DOI:** 10.1186/s13722-021-00215-z

**Published:** 2021-01-22

**Authors:** Hunter M. Puckett, Jenny S. Bossaller, Lincoln R. Sheets

**Affiliations:** 1 University of Missouri School of Medicine–Columbia, 1 Hospital Dr., MA204 Med. Sci. Bldg, Columbia, MO 65212 USA; 2 School of Information Science & Learning Technologies, College of Education, 303 Townsend Hall, Columbia, MO 65211 USA

**Keywords:** Project ECHO, Opioid use disorder, Physician preparedness, Opioids, Physician outcomes

## Abstract

Opioid use disorder (OUD) is a medical condition that has evolved into a serious and deadly epidemic in the United States. Both medical and psychological interventions are called for to end this growing epidemic, but too few health care professionals are trained to treat OUD. One proven model of training physicians and cross-disciplinary teams in treating a variety of disorders is exemplified by Project ECHO (Extension for Community Healthcare Outcomes), a collaborative tele-mentoring program in which specialists train health-care workers to treat medical conditions, especially those that affect underserved populations. This systematic review found that Project ECHO has the potential to effectively extend current services to patients suffering from OUD, but that there is also a gap in knowledge regarding this type of training. The articles that we reviewed all presented evidence that Project ECHO improves healthcare provider preparedness to treat OUD, especially in regard to improving knowledge and self-efficacy.

## Introduction

Opioid use disorder (OUD) is an ongoing problem worldwide, especially in the United States. Opioid use disorder is the psychological and physical dependence on opiates, a substance found in many prescription pain medications and some illegal drugs such as heroin. According to the Centers for Disease Control [2, 13], more than 702,000 people have died from a drug overdose between 1999 and 2017. In 2017 alone, around 70,000 people died from drug overdoses, and 68% of those deaths involved the use of prescription or illicit opioids. On average, 130 U.S. citizens die every day from an opioid overdose [14].

In 2016, the national rate of opioid-related hospitalizations was 297 per 100,000 population [3], but access to treatment services was very regionally dependent, due in part to shortages of substance-disorder psychiatrists and other physicians with a DATA-2000 waiver allowing them to prescribe buprenorphine [12]. The number of primary-care providers and other clinicians who have obtained these waivers has doubled in recent years [3], as those providers may be best situated to benefit from ECHO programming focused on OUD. Methadone is not covered by the DATA-2000 waiver and is only allowed through specially licensed opiate treatment programs. Naltrexone does not require a waiver to prescribe.

In addition to the many lives that are impacted or lost as a consequence of opioid misuse, the disease presents a large economic burden. It is estimated that opioid misuse in the United States costs victims and the government 78.5 billion dollars every year. This includes the costs of health care, lost productivity, treatment, and criminal justice involvement [14]. Compounding the problem is that many U.S. physicians are not confident treating OUD, or do not have or often use the required Medication for Opioid Use Disorder (MOUD) waiver to treat patients with buprenorphine [4].

Better training for interdisciplinary healthcare teams for OUD is needed to help control the opioid epidemic. Project ECHO (Extension for Community Healthcare Outcomes) is one educational model that could help reach physicians and others serving patients in underserved areas [15]. Project ECHO is a case-based continuing education model that is designed to enhance the capacity of community providers (typically primary care providers) in treating special conditions. The model has been widely adopted with positive results that demonstrate its utility in reaching people living in places with reduced access to health and mental health providers [9, 16, 17]. As documented by the ECHO Institute [15], 129 different OUD-focused ECHO programs have been implemented nationwide, and ECHO programs are among the drivers of increased buprenorphine waiver trainings. The ECHO Institute implemented a national program, but several states and regions have implemented additional initiatives.

Project ECHO is not traditional telemedicine. Telemedicine was designed for a physician to treat a patient directly. Project ECHO is a widely applicable, case-based tele-mentoring program that connects learners with experienced specialists in order to improve services. The learner is able to implement new services following participation in the program.

The objective of this study is to systematically review the current literature on the effectiveness of Project ECHO on OUD physician healthcare outcomes. One of the goals of Project ECHO is to improve physician preparedness, especially in rural locations where primary doctors are not trained to care for complex conditions. Usually, a specialist is required to care for a condition such as OUD [1]. In rural locations, specialists are hard to find. As a result, patients must travel long distances to receive the care that they need. Project ECHO aims to prevent this conflict. Because Project ECHO is a promising new model for training physicians to treat medical conditions, we conducted a systematic review of the evidence regarding current Project ECHO interventions for OUD. Table 1 shows the conceptual model for this systematic review in PICO format.

**Table 1:** Conceptual model for the systematic review in PICO format

P—Problem	Physicians who lack knowledge treating OUD
I—Intervention	Project ECHO for OUD treatment
C—Comparison	Pre-participation self-reported preparedness, including knowledge and self-efficacy
O—Outcomes	Post-participation self-reported preparedness, including knowledge and self-efficacy

## Materials and methods

### Review protocol

The review protocol for this systematic review was not registered in advance of publication.

### Date last searched

The final search was conducted in PubMed and Scopus on Tuesday, November 12, 2019.

### Data sources and searches

We followed the Preferred Reporting Items for Systematic Reviews and Meta-Analysis (PRISMA) recommendations in conducting this systematic review [11]. The keyword search included all types of articles from any year in Scopus and PubMed, limited to English language, all types of studies from any year (see Table 2 for search strategies). Studies were determined eligible for inclusion in this systematic review based on screening of the articles. Additional studies were found by searching the bibliographies of the articles located in PubMed. A complete breakdown of inclusion and exclusion criteria is shown in Table 3.

**Table 2:** Search strategies

Database	Search term	Results	Articles selected	Notes
PubMed	(project echo or extension for community health outcome* OR extension for community healthcare outcome*) AND (oud or opioid use disorder or OUD or opiate use disorder OR substance abuse OR opioid abuse or opiate abuse or substance use disorder or substance use disorder)	22	7	
Scopus	(Project ECHO or extension for community health outcome or extension for community healthcare outcome or extension for community health care outcome) and (oud or opioid use disorder or opiate use disorder or opiate use disorder)	3	0	(All relevant documents were duplicates from PubMed search)

**Table 3:** Inclusion and exclusion criteria

Criteria	Inclusion	Exclusion
Time period	Any	–
Language of article	Any	–
Geographical location	Any	–
Patient/physician demographics	Any	–
Study design	Observational studies and self report studies	Systematic reviews, editorials, and others
Disease addressed	Opioid Use Disorder (OUD)	Any other disease
Intervention models used	Project ECHO facilitated	Any other telemedicine program or traditional intervention

### Study selection

The authors independently screened titles and abstracts for eligibility. Both experimental and observational studies were included in the review that were deemed eligible via screening.

### Data extraction and quality assessment

One author (HP) extracted data from the included studies. The following data were extracted from the studies, if available: study design, location, duration of Project ECHO sessions, sample size, and outcomes stated by the authors of the studies. Each article was further evaluated for its contributions to current practice.

## Results

### Study selection

**Fig. 1:**
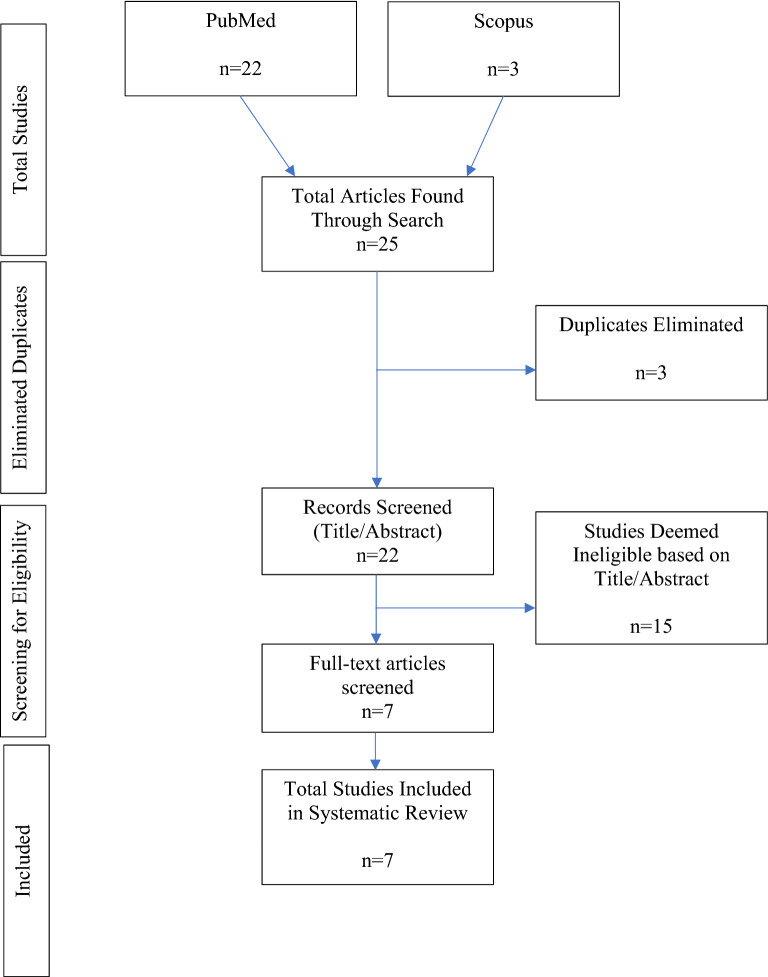
Prisma Diagram [11]

A total of 22 studies were screened and assessed for eligibility in this systematic review. After screening and assessing the 22 studies, 7 were included in the review. False hits and irrelevant articles, as well as those that fell outside of the scope of this review, were excluded. Figure 1 shows how many documents came from each search and how many were excluded at each step.

### Study characteristics

The included studies are referenced in APA citation format below, with a summary of the relevant results from each journal article.

### Results and evaluations of individual studies

Katzman et al. [5]

#### Results of study

A Project ECHO course was offered on seven different dates. 1315 clinicians across country attended, and 1079 gave consent (completed a pre-course survey) to participate in the study (92% from IHS, tribal, or urban AI/AN programs). 90% of all clinicians had prescriptive authority to treat OUD. Of 1079 participants, 909 said the survey was useful, comprehensive, interactive, well presented, and convenient.

818/1079 of respondents said that ECHO training sessions should be shorter, more frequent, adaptable, and include more details on opioids. Some expressed frustration with technical issues encountered during the training sessions. The mean scores before participating in an OUD ECHO were 8.6 out of 12 for “knowledge” and 3.7 out of 6 for “self-efficacy”; after, the mean scores rose to 10.1 out of 12 for “knowledge” and 4.4 out of 6 for “self-efficacy”. The survey provided self-reporting on knowledge of OUD and self-efficacy in providing treatment for OUD.

#### Evaluation

In this study, clinicians who attended ECHO sessions filled out a survey that measured how they believed the training impacted their knowledge of OUD and their ability to treat OUD. However, their actual gain in knowledge and self-efficacy were not tested. Therefore, it is impossible to tell if physicians who attended this training actually became better prepared to treat OUD. What can be concluded from this study is that participants felt more prepared after their ECHO training than before, indicating that Project ECHO might have had a positive impact on clinicians’ ability to treat patients with OUD, but no actual outcomes were measured [6].

#### Results of study

This study reports on an innovative, multimodal model of services to treat patients with OUD. Training included ECHO training for medical interventions, emergency medical training, and peer-based support. The authors report that the hub and spoke model of ECHO training served to connect the members of medical and psychiatric teams for more rapid, comprehensive service provision. Prior to implementation wait times were sometimes more than two weeks, and after implementation wait time was reduced to 24–48 h, which increased initial program adherence. In this study, the Hub was a comprehensive opioid treatment program in a psychiatric hospital, and the Hub clinic provided support to 12 affiliated primary care, emergency care, pain clinic, psychiatric sites, as well as outreach and bridging services to jail, drug court, and related authorities. This comprehensive method of communication and transfer resulted in shorter wait times for treatment. The majority of the patients (166/180) began MOUD upon intake to the program.

Of 121 Project ECHO participants, all of whom were primary care physicians, reported:

- Increased knowledge of evidence-based OUD management (n = 109/121, 90%)
- Decreased sense of professional isolation (n = 101/121, 83%),
- Self-reported improvement in ability to provide MOUD to patients with OUD (n = 107/121, 88%).

In addition, the training provided virtual DATA-waiver training (a requirement of the Substance Abuse and Mental Health Services Administration to prescribe or dispense buprenorphine) to 41 physicians in the state [18].

#### Evaluation

In this study, patient outcomes that resulted from physicians who received OUD training were measured. The OUD ECHO trained primary care physicians diagnosed a total of 180 patients with OUD in its first six months. Self-report measures were used in this study in attempt to gauge how the participants felt post-ECHO training. This program found program retention rates consistent with other studies, but the authors said that the method holds promise for improved services as they continue to expand the program.

The important conclusions to draw from this study are:

1. Wait times decreased, meaning Project ECHO had a direct effect on the ability of patients with OUD to receive treatment.
2. 41 physicians virtually received DATA-waiver training. This should also reduce patient wait times, as there are more physicians that are now certified to treat prescribe treatment for OUD.
3. ECHO training was implemented alongside a peer-based recovery support system, in order to help individuals adhere to treatment plans. The comprehensive nature of this program is designed to tackle all aspects of the disease, and is also designed to be scalable and adaptable to other locations [8].

#### Results of study

The researchers were interested in finding whether participants incorporated continuing education offered through ECHO into their practice. Following each ECHO session, participants were asked about how they would apply their case discussions. 104 physicians participated in the sessions, and 41 participants submitted a total of 299 post-session surveys. Not all respondents answered each question. 254/295 (85%) of respondents reported that they had learned something new from others’ cases, and 231/249 (93%) responded that the information they learned would be useful. 65/84 (77%) of those who presented a case changed their patient care plans as a result of the ECHO. 70/81 (86%) gave the training a rating of 5/5, judging the “quality of input” they received from Project ECHO. Participants reported that they learned new knowledge related to diagnosis, behavioral/medical interventions, and resources outside their practice, and increased self-awareness in patient interactions to reduce stigma.

#### Evaluation

This data used in this study came in the form of survey responses. A majority of respondents felt that they learned something new and that the information presented in the ECHO sessions would be useful in the future. Again, like the first study analyzed in this paper, only self-assessment data was used. Therefore, whether or not participants actually gained knowledge with regard to OUD is unknown. The important thing to take away from this study is that 93% of participants felt that the information presented in ECHO training way useful. It can be concluded that ECHO is conveying information that gives healthcare professionals the opportunity to improve their ability to treat patients with OUD [8].

#### Results of study

This study reported that since 2008, over 950 patient cases have been presented and 9000 h of CME credit has been awarded through the University of New Mexico’s Health Sciences Center. Each session consists of a 2-h case presentation, with discussion facilitated by a physician specializing in OUD, a psychiatrist, a social worker, a psychiatric nurse, and a community health worker. On average, 147 new participants join every year, and since Jan 1, 2010 654 unique participants attended at least one ECHO clinic, and 285 people attended more than one ECHO clinic.

Since 2006, only 36 NM physicians were listed as buprenorphine waivered, but post ECHO training, 375 physicians in NM are now waivered. This ranks 4th in U.S. per capita. The format of ECHO training is especially appealing to rural areas such as those within New Mexico, where providers have fewer opportunities to collaborate with specialists in their setting.

#### Evaluation

The importance of this study is the increase in buprenorphine waivered physicians. However, it is impossible to tell how many became waiver certified as a direct result of ECHO. This means that more physicians are able to treat patients with OUD, which will increase the opportunities for patients with OUD to receive treatment [10].

#### Results of study

This study reports that since May 2019, 9 ECHO sessions were offered, and 30–50 providers attended each session. Preliminary results suggest that more providers are obtaining waivers, but would like more training to treat more patients.

#### Evaluation

This study states that preliminary results suggest that more providers are becoming waivered. No real impact of ECHO training was proven. This article is weak in supporting the impact of ECHO on physician preparedness to treat OUD [16].

#### Results of study

A total of 24 primary physicians and clinical staff members from 13 different clinics were registered to attend a 12-week Project ECHO case-based OUD treatment curriculum. The facilitators kept participation logs and participants were interviewed about their experience in the program. The authors report that primary care physician (PCP) attendance to ECHO sessions was variable, with higher rates of participation among prescribers (mean/SD rate of 5.9/3.6) than non-prescribers (mean/SD rate of 2.6/3.1), and higher rates of attendance during early weeks of the program. Despite relatively low rates of participation, interviews indicated that the program was successful because of the ease of access of the sessions. One participant explained: “I like it online because there’s no travel involved.... I can easily jump out and jump back in as issues come up around the clinic” (n.p.).

Medical providers expressed support for the opportunity to engage with content experts through interactive case presentations. Participants emphasized the value-added effects of participating in the ECHO sessions. The most notable engagement challenge reported by participants was the burden of competing demands on time and availability. All providers and staff indicated that the one-hour ECHO session is an appropriate duration, but they consistently cited the need to prioritize patient care over other activities. The other primary barrier participants reported pertains to the degree to which clinic leadership supported devoting resources to starting or expanding [MOUD], including time for attending the ECHO sessions. Providers noted that willingness leadership is required to integrate MOUD in the clinic.

#### Evaluation

The main draw from this study is how Project ECHO could be improved. Since time and availability was one of the main issues reported by participants, recording ECHO sessions to be viewed at a later time or date could solve this issue [19].

#### Results of study

ECHO program consisted of 16 weekly one-hour sessions, where 53% of participants attended 10/16 ECHO sessions. The average attendance was 9/16 weeks. The mean scores before participating in an OUD ECHO were 51.4% for “knowledge” and 58.1% for “self-efficacy”; after, the mean scores rose to 58.4% for “knowledge” and 73.2 for “self-efficacy”. The number of participants prescribing buprenorphine rose from 7 to 10.

#### Evaluation

Again, self-report data was analyzed in this study. While participants feel ECHO improved their knowledge and self-efficacy, no real test was administered to measure this.

A synthesis of the results from all the studies included in this review is shown in Table 4.

**Table 4:** Synthesis of the systematic review results

Article author	Time period	Number of participants	Self-efficacy improvements?	Increased knowledge how to treat OUD?	Waivers obtained?	Health outcomes?	Qualitative outcomes?	Other outcomes?
Katzman et al. [5]	7 Five-hour long courses on seven different dates	1315 attended, 1079 gave consent to participate in the study	Yes	Yes	No	No	No	N/A
Kawasaki et al. [6]	Not specified	Varies based on survey	Yes	Yes	Yes	No	No	Shorter wait times for patients and decreased sense of professional isolation for physicians
Komaromy et al. [7]	Two-hour session every week	Varies based on survey	No	Yes	No	77% of physicians changed patient care plan	No	ECHO participants rated input 5/5
Komaromy et al. [8]	Two-hour sessions every week	654 unique partic-ipants attended at least one clinic	No	No	Yes	No	No	N/A
Miele et al. [10]	One-hour session every month	30–50 per session	No	No	Yes (*prelim. results*)	No	No	N/A
Salvador et al. [16]	12 one-hour sessions for 12 consecutive weeks	24 partici-pants from 13 primary care clinics	No	No	No	No	Support for the opportunity to engage with content experts	Ease of access to ECHO sessions was reported, participants emphasized the value-added effects of participating in the ECHO sessions
Tofighi, B., et al. [19]	One-hour sessions every week for 16 weeks	17	Yes	Yes	No	Increased prescribers of buprenorphine	No	Feedback for improving ECHO program included archiving recordings of sessions, clinical shadowing of buprenorphine providers, and increasing the involvement of non-physician clinical staff

## Discussion

The findings of this systematic review have shown that Project ECHO has the potential to improve OUD education among previously untrained or undertrained physicians and other health care professionals. Project ECHO was first launched in 2003 to increase access to hepatitis C treatment, and has since expanded to 220 teams in 31 countries addressing more than 100 conditions, including OUD. However, a study by the Rand Corporation found that building capacity to evaluate ECHO outcomes is a critical unmet need. There was no contradictory data among the journal articles reviewed and included in this systematic review. Additional research is needed that focuses on the efficacy of the program in relation to its effects on individual physicians.

We already knew that Project ECHO is effective in reaching a large number of health care providers in a timely and productive manner. What we did not know about Project ECHO was its effectiveness or whether or not it had positive impacts on those who participated in the program. This systematic review has shown preliminary results in its effectiveness regarding physician preparedness to treat OUD.

Since many of the studies analyzed in this study were studies that involved self-report data, it is impossible to tell how Project ECHO really changed the ability of physicians to treat OUD. This low-quality data is why more research needs to be done with regards to OUD and ECHO. This is discussed in the conclusion.

There were some limitations when writing this systematic review. Only studies freely accessible online or available through the University of Missouri’s databases were included in the study. Every effort was made to minimize source bias. We attempted to avoid database bias by searching the two largest, most relevant databases for our review, PubMed and Scopus. We acknowledge that publication is almost always present, as research studies with negative or unintended outcomes are rarely published, especially if they were funded by an outside influential organization.

## Conclusions

At this point Project ECHO shows promise in expanding treatment of OUD, though there is a need for further studies. ECHO participants reported that ECHO classes are easy to access and added value to their medical education, though many expressed frustrations with technical issues encountered during the training. All of the articles reviewed in this paper indicate that Project ECHO improves physician preparedness to treat opiate use disorder. Knowledge and self-efficacy were the two areas where physicians improved the most. Many physicians also obtained buprenorphine waivers, allowing patients with OUD to prescribe drugs they need to treat OUD more effectively. This evidence indicates that Project ECHO should be used more widely to expand patients’ timely access to OUD treatment.

Research is still needed to determine the widespread health outcomes of Project ECHO on OUD. The research papers we reviewed showed positive results on physicians and other health care providers, but limited healthcare outcomes were reported. In the future, more data needs to be collected regarding Project ECHO’s long-term effectiveness. While the studies included in this review show early positive signs of Project ECHO’s potential, more research needs to be done to determine if increased physician preparedness translates to better OUD health outcomes in patients. While the reviews included in this study look at how physicians in particular are impacted by Project ECHO, little data exists that shows whether patient health care outcomes are affected. The ultimate goal of Project ECHO is to improve the quality of care for patients through the training of physicians. Therefore, research needs to be done to see if Project ECHO trained physicians increase recovery speed in patients with OUD.

Current research demonstrates that Project ECHO is beneficial, but further research is needed to confirm the effectiveness of Project ECHO, especially in regard to specific medical conditions such as opioid use disorder.

## Data Availability

Data is publicly available in PubMed and Scopus. See Table 2.

## Abbreviations

ECHO: Extension for Community Healthcare Outcomes
MOUD: Medications for Opioid Use Disorder
NYC: New York City
OUD: Opioid use disorder
U.S.: United States
